# Nanowall Textured Hydrophobic Surfaces and Liquid Droplet Impact

**DOI:** 10.3390/ma15051645

**Published:** 2022-02-22

**Authors:** Bekir Sami Yilbas, Abba Abubakar, Mubarak Yakubu, Hussain Al-Qahtani, Abdullah Al-Sharafi

**Affiliations:** 1 Mechanical Engineering Department, King Fahd University of Petroleum and Minerals, Dhahran 31261, Saudi Arabia; abba.abubakar@kfupm.edu.sa (A.A.); g201705190@kfupm.edu.sa (M.Y.); qahtanih@kfupm.edu.sa (H.A.-Q.); alsharafi@kfupm.edu.sa (A.A.-S.); 2 IRC for Renewable Energy and Power, King Fahd University of Petroleum and Minerals (KFUPM), Dhahran 31261, Saudi Arabia; 3 K.A. CARE Energy Research and Innovation Center, Dhahran 31261, Saudi Arabia; 4 Engineering Faculty, Turkish Japanese University, Istanbul 34906, Turkey

**Keywords:** droplet impact, hydrophobic nanocells, silicon wafer, nanowalls

## Abstract

Water droplet impact on nanowires/nanowalls’ textured hydrophobic silicon surfaces was examined by assessing the influence of texture on the droplet impact dynamics. Silicon wafer surfaces were treated, resulting in closely packed nanowire/nanowall textures with an average spacing and height of 130 nm and 10.45 μm, respectively. The top surfaces of the nanowires/nanowalls were hydrophobized through the deposition of functionalized silica nanoparticles, resulting in a droplet contact angle of 158° ± 2° with a hysteresis of 4° ± 1°. A high-speed camera was utilized to monitor the impacting droplets on hydrophobized nanowires/nanowalls’ textured surfaces. The nanowires/nanowalls texturing of the surface enhances the pinning of the droplet on the impacted surface and lowers the droplet spreading. The maximum spreading diameter of the impacting droplet on the hydrophobized nanowires/nanowalls surfaces becomes smaller than that of the hydrophobized as-received silicon, hydrophobized graphite, micro-grooved, and nano-springs surfaces. Penetration of the impacted droplet fluid into the nanowall-cell structures increases trapped air pressure in the cells, acting as an air cushion at the interface of the droplet fluid and nanowalls’ top surface. This lowers the droplet pinning and reduces the work of droplet volume deformation while enhancing the droplet rebound height.

## 1. Introduction

Impacting liquid droplets on hydrophobic surfaces finds various applications in areas of heat transfer, coating, fire extinguishing, inkjet printing, fuel injection, metal quenching, self-cleaning, drug delivery, and so on [1,2,3,4,5,6]. As the texture and chemical properties of the impacting surfaces change, the dynamic characteristics of the droplet alter [7,8,9]. This yields variation of droplet rebound height, spreading, and retraction rates; hence, the droplet spreading behavior is strongly influenced by the wetting state of the hydrophobic surface. The droplet contact angle and droplet spreading behavior are strongly affected by micrometer and nanometer size roughness; hence, increasing surface roughness can increase the contact angle depending on the surface tension of the droplet fluid [8,9,10]. The maximum spreading diameter and contact time on the hydrophobic surface decreases as the contact angle increases, while the rebounding height increases. This is attributed to the increase in the energy dissipation against pinning effects with the decrease in wetting diameter [7]. In addition, the behavior of the impacted droplet changes with the topology of the hydrophobic surface. In general, three distinct behaviors can be observed for the impacting droplets. These include non-bouncing, complete bouncing, and partial bouncing regimes, which become particularly observable on the micro-pillared hydrophobic surfaces [11]. Depending on the pitch size and the impact velocity, the droplet can penetrate micro-pillared gaps, affecting the distinct droplet behavior on the impacted surface. For example, rebounding droplet shapes change as the surface topology changes. In this case, impacting droplet kinetic energy dissipation over the impacted surface plays a major role in droplet shapes upon the rebounding [12]. This is because water hammer pressure and penetration time change during the impact’s spreading and recoil stages [13]. Hence, the patterns created on the impacting surface influence the droplet behavior. This becomes particularly important for biophilic wetting state surfaces. Due to the shear rate at the droplet–solid interface, the frictional force significantly changes the droplet behavior on the surfaces [14]. The contact time and spreading lengths of the droplet can be considerably reduced, and the rebounding height improved with the impact on specially designed multi-ridge structure (with 20–30 ridge angles) or micropillar surface created by an array of nanotubes due to the combined effect of center-drawing recoil, weaker wettability of drops, and their penetrating ability into the valleys between surface protrusions [15,16]. The contact time of impacting droplets on carbon soot nanocoating also becomes shorter due to the irregular hierarchical nanoparticle (or flower-like) networks formed on the surface [17,18]. Similarly, a fourfold reduction of contact time results for a droplet impacting a hydrophobic surface with micron-scale posts decorated with nanotextures. This is attributed to the pancake bouncing behavior whereby the droplet rebounds without retracting. The combined influence of pinning forces and the rectification of capillary energy stored in the penetrated liquid contributes to the increase in the kinetic energy of the rebounding droplet [19]. It is further shown that, by designing the surfaces to have tapered micro/nanotextures that act as harmonic springs, further reduction in contact time is possible; however, for such a case, the contact time becomes independent of the impacting velocity. Furthermore, introducing the unidirectional micro/nano strips on the hydrophobic surface allows non-uniform spreading and retraction of the droplets. Hence, the non-axisymmetric behavior of the impacted droplet can be explored to extend the droplet in certain directions [20]. Hence, contrary to the results of established theories, the contact time is found to reduce for a droplet bouncing from an asymmetrically patterned (flat) hydrophobic surface compared to that of an axisymmetric one mass redistribution and center-assisted recoil action. Consequently, the droplet rebounding kinetic energy becomes increased [21]. A similar study also shows that the asymmetric bouncing of impacting droplets on convex/concave hydrophobic surfaces results in shorter contact time due to asymmetric momentum exchange and mass redistribution that allows for preferential fluid pumping around the drop rim [22]. As the hydrophobic surface has a curvature rather than micro/nano-strips, breaking up the impacted droplet becomes unavoidable, particularly at large Weber numbers. In addition, the curvature effect hinders the rebound of the droplet upon impact. In this case, the spreading rate reduces with increasing curvature of the hydrophobic surface [23]. The irregular texturing of surfaces by laser ablation can also create hydrophobic and superhydrophobic surfaces. Impacting droplets demonstrate different behavior on laser textured surfaces than those hydrophobic surfaces having hierarchical texture patterns. The shorter recoiling and longer rebound times are observed for laser textured surfaces [24].

The surface characteristics play a critical role in droplet behavior, particularly for the impacting droplets. In some cases, such as biomedical applications, the droplet can be composed of the impacted fluid, and the droplet spread is expected to be small upon impact to avoid the large spreading areas. In general, the hydrophobic surfaces have low surface free energies and hierarchical textures topology such that the droplet spread can be minimized after the impact. Hence, the hierarchical texture topology with unique features becomes critical in avoiding the spread of the infected fluids. Furthermore, as the surface texture is formed from locally isolated (closed) cells, impacting droplet fluid penetrates the isolated cells. In this case, the droplet fluid compresses the trapped air in the cells during the impacting–spreading periods. As the droplet spreading cycle is completed, the compressed air in the cells can push the droplet fluid from the surface while creating an air cushion over the surface interface. This can modify the retraction and rebounding properties of the impacted droplet. As the texture height increases, the large penetration of impacting droplet fluid occurs within the texture pillar gaps, increasing the three-phase wetted length on the textured surface. In addition, the deep penetration of droplets into the texture suffers from the droplet liquid pinch-off [25]. This can change the droplet pinning behavior on the impacted surface while altering the droplet stretching and retraction cycles. On the other hand, hydrophobizing the surface toward creating textures consisting of long pillars with sharp edges is challenging. These textures can also possess isolated partially closed cells, such as nanowires/nanowalls, which can be formed on silicon-wafer surfaces [26]. Introducing functionalized nanoparticles on such pillar surfaces can create the hydrophobic wetting state on the texture [26]. Recently, it has been shown that the silicon-wafer surface structured with vertically aligned silicon nanowalls becomes near superhydrophobic with excellent water-repellent properties. In contrast, bunched nanowalls lead to hydrophilic behavior [27,28]. However, investigations about impacting droplet characteristics on such nanowall-textured surfaces are rare to find in the open literature. Consequently, further investigations about the droplet-impact behavior on nanowall textured hydrophobic surfaces having large pillar heights with sharp edges are needed.

Depending on the morphology of impacting hydrophobic surfaces, the droplet volume [20], the curvature of the surface [23], and irregular surface patterns [24] influence the impacting characteristics. Although the droplet impact on hydrophobic surfaces has been reported extensively in the previous studies [20,23,24], the influence of surface texture, composed of closely spaced nanowires/nanowalls cells, on the impacting droplet characteristics is left for future study. Creating a combination of nanowires/nanowalls on surfaces requires the control process of texturing. Moreover, nanowires/nanowalls are nanosize structures with relatively sharp edges, and the hydrophobicity of such textured surfaces becomes challenging [26,27,28]. In addition, such surface textures provide different spreading, retraction, and rebounding characteristics of the impacting droplet than those impacting on plain or soft hydrophobic surfaces. The spreading and retraction of the droplet on the impacting surface are critically important for a droplet transition period, particularly in icing, drop-wise condensation, droplet evaporation, and similar applications. Consequently, the investigation into the droplet-impact characteristics on hydrophobized nanowires/nanowalls becomes essential. The present study examined the impact of droplet dynamics on hydrophobized silicon nanowires/nanowalls. Silicon wafers were used to generate silicon nanowires/nanowalls on the surfaces through the chemical etching process in line with that reported in the early work [26]. The surface morphology and textures were analyzed via analytical methods, and wetting states are evaluated by using the contact-angle measurement technique. The droplet spreading, retraction, and rebounding behaviors were investigated for different impact heights and droplet sizes by utilizing the high-speed recorded data.

## 2. Experimental

A P-type <100> silicon was utilized for etching towards creating nanowires/nanowalls textures on the surface. In the etching process, a mixture of AgNO_3_ (99.8%), H_2_O_2_ (30% in water), H_2_SO_4_ 98%, and HF (48%) was incorporated as the etchant. The p-type wafers were prepared in 20 × 20 × 1 mm^3^ size. They were cleaned with piranha mixture (H_2_O_2_: H_2_SO_4_ (1:1 *v*/*v*)) and later sonicated for 12 min. The samples were treated with HF/AgNO_3_ solution with the volume ratio of 1/3 for 30 min, and they were enclosed by silver nanoparticles. The etching was initiated by using HF/H_2_O_2_ 5 M/ 30% solution as an etchant. The etched surfaces were dried under a nitrogen environment. Silver residuals on sample surfaces were removed via immersion of the samples in H_2_O: HCl: HNO_3_ (1:1:1 *v*/*v*/*v*) solution for 45 min, and, later, the sample surface was dried in a nitrogen ambient. The texture of the etched surfaces was analyzed by using a scanning electron microscope (Jeol 6460, JEOL, London, UK) and an atomic force microscope (Flex-Axiom, Nanosurf, Bracknell, UK). Functionalized nano-silica particles were deposited on the etched and as-received wafer surfaces via the dip-coating process. In the functionalizing cycle, the procedure developed in the early work was adopted [29]. In this case, tetraethoxysilane (TEOS), octyltriethoxysilane (OTES), ethanol, and ammonium hydroxide were used in the functionalizing process. A Goniometer (Kyowa, model DM 501, Tokyo, Japan) was utilized to evaluate surface wetting states through contact-angle measurements. The Goniometer had automatic water dispensing and a droplet recording system. The contact angles were measured by adopting the statistical contact-angle analyses (high-precision drop shape analysis (HPDSA)), as presented in the early work [30].

Distilled water was used to create droplets of volumes 10, 20, and 30 μL, and impact heights of the droplets were set at 10, 20, and 30 mm in the experiments. A high-speed camera (Dantec Dynamics SpeedSense 9040, Hovedstaden, Denmark) was utilized to record the impacting droplets. The recording was carried out at 5000 frames-per-second (fps) with a megapixel resolution (1280 × 1000). The size of the pixel was 14 µm × 14 µm during the recording. A tracker program was used to extract the high-speed data records and evaluate the droplet speed and relevant droplet impact characteristics (spreading, retraction, droplet height, rebounding, etc.). The uncertainty analysis was conducted to estimate the experimental uncertainty involved in the measurements. The uncertainty (±𝑢) was calculated by using (1) data obtained from the tracker program, such as impacting droplet velocity, spreading and retraction rates, droplet height, rebounding height/velocity, and similar things; and (2) errors pertinent to the measurements in pixel variations. The extracted data-confidence level based on repeats was found to be 96%, while the error was estimated as about 3% based on the Gaussian distribution. The uncertainty (*σ_u_*) was as follows [31]:

(1) $$\sigma_{u}=\sqrt{\int_{x_{o}}^{x_{n}}{\left(x-\mu_{e}\right)}^{2}p\left(x\right)dx}$$

where *µ_e_* represents the mean value of *x, n* is the number of data points, and *p(x)* corresponds to the probability function, which was extracted from the correlation plane by using all the data points. The resulting function was closed-fitted in a Gaussian form toward assessing the diameter of the function. The uncertainty was evaluated by adopting the Gaussian fit, and the final evaluation was normalized over the total pixel points used in the cross-correlation. The bias uncertainty was evaluated as 0.02 pixels based on the small peaks in the distribution function. The uncertainty was estimated at 3%.

## 3. Results and Discussions

Impacting liquid droplets on hydrophobized silicon nanowire/nanowall surfaces were examined, and the behaviors of the different size droplets in terms of spreading, retraction, and rebounding were analyzed. The high-speed camera and tracker program were used to monitor and evaluate the motion of the impacting droplets.

### 3.1. Hydrophobic Silicon Nanowires/Nanowalls Texture

**Figure 1:**
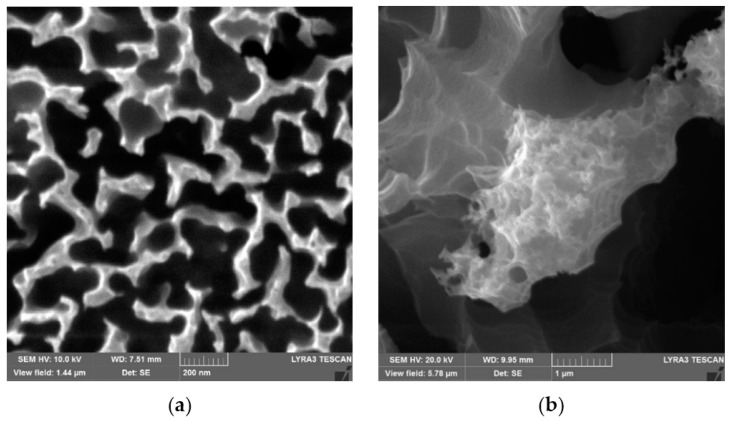
SEM images of top surfaces of nanowires/nanowalls: (**a**) combination of nanowires and nanowalls (nanowires are marked in circle); (**b**) nanowalls have porous-like texture on the top surface.

Silicon wafers of p-type (Si <100>) are etched chemically toward creating silicon nanowires/nanowalls on the surface. Figure 1a,b shows the top view of the etched wafer surface. Nanowalls are formed simultaneously with some nanowires on the wafer surface during the etching. The nanowalls have large edges in the top region compared to those of the nanowires; that is, controlled etching does not produce complete nanowire structures over the wafer surface, but the mixture of nanowires and nanowalls is created (Figure 1a). The mixture composition of the nanowalls and nanowires is related to the etching duration and concentration of the etchant [32]. Hence, preferentially oxidized silver nanoparticles result in the non-uniform etching of silicon wafers during the etching process. Since silver-nanoparticles-assisted etching is carried out in the solution of HF, and an oxidant (H_2_O_2_; O_2_ present in H_2_O), some regions on the silicon wafer surface are not completely etched. This feature appeared as jointly connected nanowires contributing to the formation of silicon nanowalls. In this case, the transient response of the surface to the chemical reactions can probably vary locally over the tens of nanometers scales on the sample surface during the etching process. This alters the geometry of the nanowalls on the surface, even though the distribution of the nanowalls shows a hierarchical topology. It is worth mentioning that, to create surface topology composed of the mixture of nanowires and nanowalls, many etching tests are conducted, adopting the different concentrations of the etching solution and the etching periods; that is, some of the solution concentration and etching cycles do not result in nanowires and nanowalls on the silicon wafer surface. In addition, the initial etching process involved 5 M HF 0.02 N AgNO_3_ for 90 s of etching. In the second consecutive process, HF/H_2_O_2_ 5M/30% in H_2_O was used to etch the initially etched surfaces for an additional 11 min. The consecutive etching processes create a combination of nanowires and nanowalls on the silicon-wafer surface. The spacing between nanowalls and nanowires changes across the etched surface; however, the average spacing is about 130 nm (Figure 1a). The formation of the porous structures was also observed in the tip section of the nanowalls (Figure 1b), which are initiated during the first etching and are further developed during the second etching process. The coverage area of the porous structures is limited to the tip region of the nanowalls (Figure 1b).

**Figure 2:**
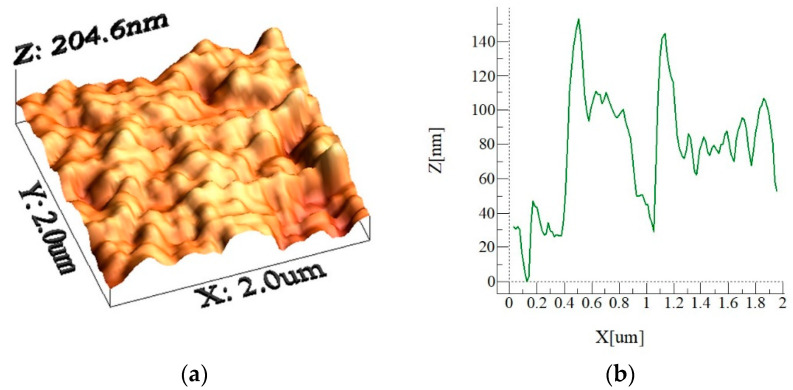
Three-dimensional AFM image of the etched silicon-wafer surface and line scan: (**a**) image of the etched surface and (**b**) line scan of the etched surface.

In addition, 3D-AFM images and line scans of the surface are shown in Figure 2. It is worth mentioning that the AFM probe is operated at the tapping mode rather than the friction mode because of the sample surface topology, which consists of texture with pillars and cavities. The AFM probe tip is made of silicon nitride with a tip radius in the range of 20 nm. The AFM image (Figure 2a) demonstrates that the surface texture has a similar pattern composed of pillars and valleys. The AFM line scan (Figure 2b) shows the multiple spikes over the surface texture. The presence of the spikes demonstrates the nanowire/nanowall combinations on the surface. In this case, the sharp spikes resemble the nanowires, while the sharp spike following the low amplitude spike corresponds to the nanowires on the etched surface. Nevertheless, the line scan of the mixture of such patterns demonstrates that nanowires and nanowalls have sharp edges.

**Figure 3:**
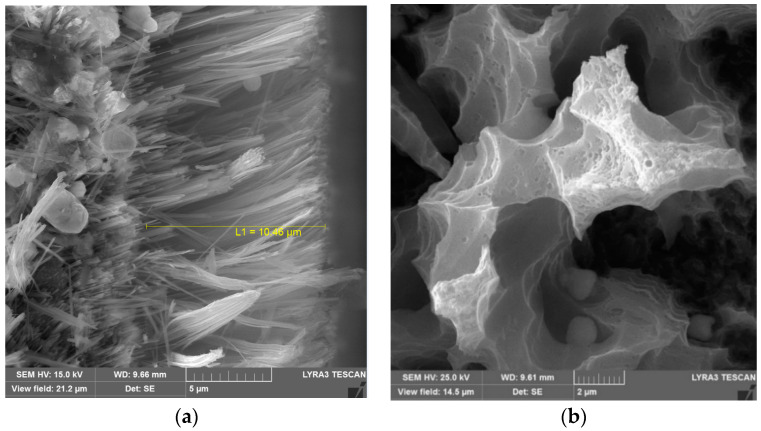
SEM microimages of side and top views of nanowalls: (**a**) nanowall side view showing height of nanowalls and (**b**) close top view of silica-particles-deposited nanowall surface.

Figure 3a depicts the side view of the etched silicon wafer. The silicon nanowires/nanowalls are almost normal to the surface, and their height extends to about 10.45 µm above the wafer surface. In addition, some of the nanowires/nanowalls have irregular shapes; that is, the thickness of the nanowires/nanowalls reduces in the top region. The irregular shapes are associated with the two-step etching process. Figure 3b depicts SEM images of the functionalized silica-particles-coated etched silicon-wafer surface. The functionalized silica particles cover almost completely over the top surface of the nanowalls, which appear as the clustered nanoparticles on the nanowall top surfaces. However, few porous sites are present on the nanoparticle-deposited surface, which is not covered by the functionalized silica nanoparticles. Nevertheless, the large area of the nanowall top surfaces is covered by the deposited particles. The contact-angle measurements are carried out to evaluate the wetting states on the particles deposited and uncoated surfaces.

**Figure 4:**
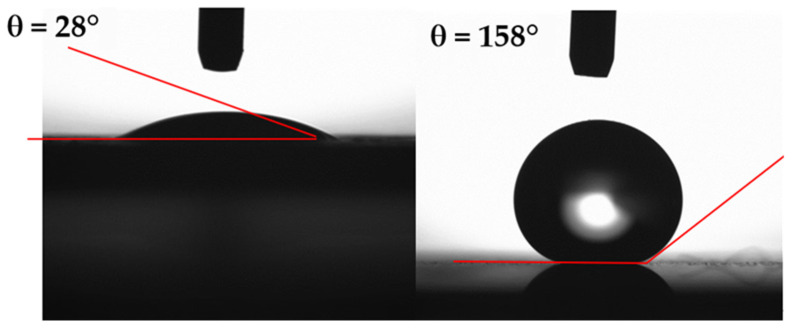
Goniometer images of a water droplet on the etched and hydrophobized etched silicon wafer.

Figure 4 shows the droplet images obtained from the Goniometer during the contact angle measurements. The contact angle of the coated wafer surface is about 158° ± 2°, and the contact angle hysteresis is 4° ± 2°. The contact-angle measurements are extended to include the uncoated etched wafer surfaces. The contact angle of the untreated (uncoated) surface is about 28.5° ± 1° with the contact-angle hysteresis of 26° ± 3°. It is worth mentioning that the high-precision drop shape analysis, as reported in the literature [30], is adopted for the contact-angle measurements. Moreover, the wetting state of the hydrophobized nanowires/nanowalls surface is also evaluated by using silicon oil, which has a viscosity of 10 cSt, density 935 kg/m^3^, and surface tension of 35 mN/m. Silicon oil spreads over the hydrophobized nanowires/nanowalls surface, while reducing the contact angle.

### 3.2. Impacting Droplet Characteristics over Nanowalls Texture

Impacting droplet suffers from spreading and retraction on the impacted hydrophobic surface. Depending on impact height, droplet size, droplet fluid properties, surface wetting state, and texture characteristics, the spreading and retraction behavior of the droplet can change. The forces influencing the impacting droplet behavior are, generally, associated with liquid pressure, droplet pinning, and interfacial shear created over the impacting surface. Since the impacted surface possesses nanowires/nanowalls, the surface texture can be considered as porous-like structures. Although these structures do not follow circular shapes on the surface, the equivalent diameter (hydraulic diameter) can resemble pore-like structures on the surface. Upon the impact, the droplet fluid can partially penetrate the spacing between nanowalls without breaking into the nanosize droplets. The analytical formulation of spreading of impacting droplets over hydrophobic surfaces was presented earlier by another study [33]; however, the study covers high Reynolds and Weber numbers. The inertial force remains critically important over the surface tension and viscous forces. Nevertheless, the droplet fluid penetration into porous-like structures for low Reynolds number impact can be approximated via the Hagen–Poiseuille’s formulation, which can take the following differential form [34]:

(2) $$d\forall ≅\frac{\pi D_{H}^{4}\sum P}{8\mu x}dt$$

where d∀ is the volume of droplet fluid penetrating a porous-like structure, *D_H_* is the hydraulic diameter of the porous site, ∑P is the total pressure of the droplet fluid penetrating porous site, *μ* is the droplet fluid viscosity, *x* is the penetration depth, and *t* is the time. It is worth mentioning that, after the penetration, it is assumed that a semispherical liquid droplet is formed in the frontal section of the penetrating fluid with a diameter the same as the hydraulic diameter of the porous site. The total pressure of the droplet fluid penetrating the porous site includes the capillary pressure, which is the Laplace pressure: *P_L_* ~ $-\frac{2\gamma cos\theta }{r_{H}}$, where *γ* is surface tension, and *θ* is liquid contact angle on the porous surface. The water hammer pressure, which is created after impact, is *P_h_* *~aρcV*, where *a* is constant, which is about 0.5 for small water droplets [35]; *ρ* is the fluid density; *c* is the speed of sound; and *V* is the droplet impacting velocity. The dynamic pressure of droplet fluid upon impact is *P_d_* ~ $\frac{\rho V^{2}}{2}$). Since the functionalized silica nanoparticles do not coat the side-surfaces of the nanowalls, these surfaces have hydrophilic characteristics, and the contact angle between the droplet fluid and the nanowall side-surface is considered to be the same as the droplet contact angle on the silicon wafer surface, which is about 33.5° ± 2°. It is worth mentioning that the capillary pressure acts opposing the droplet fluid penetration into the nanowall sites [36]. In addition, if the volume of the droplet fluid penetrating the porous site is approximated as an incremental cylinder of the porous site of the hydraulic radius, *r_H_*, then the volume of penetrating liquid yields the following: d∀ ~ $\pi r_{H}^{2}dx$, where *dx* is the incremental penetration depth of the fluid. The arrangement of Hagen–Poiseuille’s relation yields the following:

(3) $$xdx=\frac{2\pi r_{H}^{2}\left(-\frac{2\gamma cos\theta }{r_{H}}+a\rho cV+\frac{\rho V^{2}}{2}\right)}{\mu }dt$$

**Figure 5:**
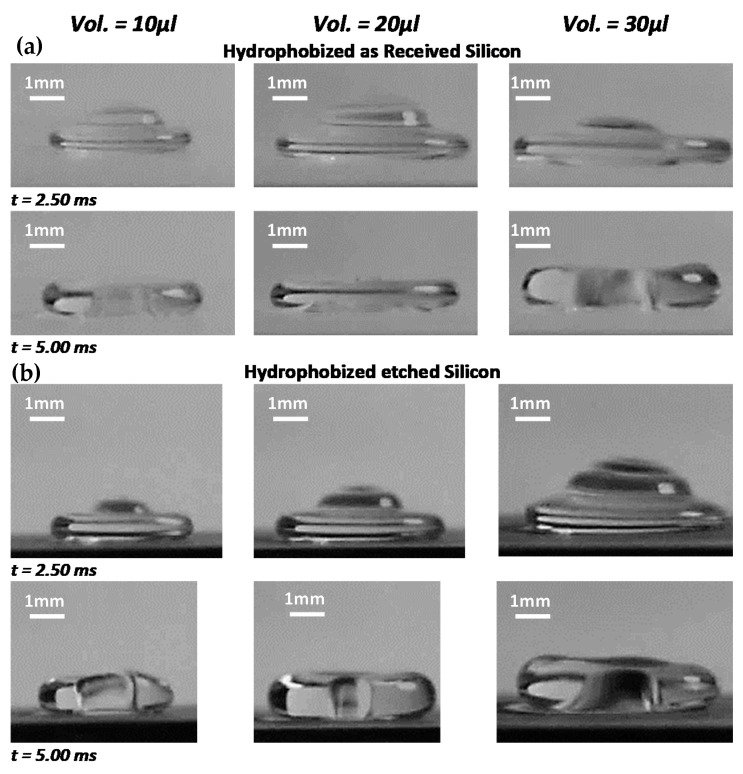
High-speed camera images of impacting droplet at different transitions on the impacted surface for (**a**) hydrophobized etched and (**b**) hydrophobized as-received silicon wafer. The droplet impact height is 20 mm.

In Equation (3), if the total pressure is replaced by the Laplace pressure, then the relation yields $xdx=\frac{2\pi r_{H}^{2}\left(\frac{2\gamma cos\theta }{r_{H}}\right)}{\mu }dt$, which becomes the same as the relation presented for the capillary penetration of liquid in the porous medium [34]. Moreover, the condition of the total fluid pressure greater than unity (*P_h_* + *P_d_* + *P_L_* > 0) results in $a\rho cV+\frac{\rho V^{2}}{2}-\frac{2\gamma cos\theta }{r_{H}}>0$ for the impacting droplet. In the limit, for *P_h_* + *P_d_* ≥ *P_L_*, the relation between the critical Weber number (*We_o_*) and the wetting state of the nanowall inner surface can be developed. Hence, the critical number, which enables the droplet fluid to penetrate the nanowall porous site, yields the following:

(4) $$We_{o}\geq \frac{8M_{l}cos\theta }{M_{l}+2a}$$

where *M_l_* depends on the droplet impacting velocity and speed of sound ($M_{l}=\frac{V}{c}$), and it remains constant for a fixed droplet impact height and the droplet fluid. Moreover, the liquid penetration depth yields the following:

(5) $$x=\sqrt{\int_{0}^{t_{p}}\frac{2\pi r_{H}^{2}\left(-\frac{2\gamma cos\theta }{r_{H}}+a\rho cV+\frac{\rho V^{2}}{2}\right)}{\mu }dt}$$

where *t_p_* is the end of the spreading period of the droplet after the impact. The liquid penetration depth into the porous site of about 65 nm hydraulic radius becomes 0.04 μm for the droplet spreading period of about 2 ms. Hence, the droplet fluid penetration into the nanopore site occurs in a small portion of the top region of the nanowalls cells (pores site). Nevertheless, the ratio of penetration depth to porous site (nanowalls cell) height is in the order of 0.003. The scale analysis can be considered to evaluate the contribution of the pressure to the droplet fluid penetration. The ratio of water hammer pressure over the dynamic pressure ($\frac{P_{h}}{P_{d}}$) is ~$\frac{2ac}{V}\;$, which yields the values much greater than unity. Hence, in the early stage of the impact, the water hammer pressure becomes more important than the dynamic pressure of the droplet in terms of the droplet fluid penetration into the nanowall pores sites. On the other hand, the nanowall pore sites have sharp edges, which modify the pinning of the impacting droplet during spreading and retraction on the surface. The texture composition of the hydrophobized wafer surface is composed of a mixture of heterogeneously coated nanowalls and the gaps filled with air; therefore, the wetting state of the surface possesses the Cassie–Baxter state. The apparent contact angle of the coated surface ($\theta^{*}$) can be expressed as $cos\theta^{*}=\varphi_{s}\left(cos\theta +1\right)-1$, where $\varphi_{s}$ is the solid fraction of the textured wafer surface. Moreover, the droplet receding and advancing angles play an important role in the droplet pinning during the droplet transition on the impacting surface. In line with the Cassie model [37,38], the receding contact angle yields $cos\theta^{*}=2\varphi_{s}-1$. Hence, closely spaced nanowall pore sites influence the surface droplet pinning. The force of pinning ($F_{pin}$) of the droplet across a single pore site can be approximated as follows:

(6) $$F_{pin}\sim \pi D_{H}\gamma cos\theta^{*}$$

where *D_H_* is the pore site hydraulic diameter. It is worth mentioning that the top surface of the nanowalls has a rough morphology, and the apparent contact angle is used. Therefore, the droplet liquid pinning force across the porous surface (composing of nanowalls) can be approximated as $\sim \sum n\pi D_{H}\gamma cos\theta^{*}$, where n is the number of pore sites across the impacting surface wetted by the droplet fluid during the spreading and retraction. Since the ratio of the area of the nanowall pores over the total etched surface area is about 3%, the pinning force becomes considerably large. This is because of the large number of pore sites across the projected area of the sample surface even though the wetting length (perimeter) of a single pore is considerably small (~800 nm). Consequently, the spreading diameter of the droplet on the hydrophobized textured surface becomes smaller than the hydrophobized as-received smooth silicon surface. This can be observed from Figure 5, in which optical images of the impacting droplet on textured and as-received smooth hydrophobized silicon-wafer surfaces are shown for different spreading phases.

### 3.3. Work of Dissipation on Droplet Spreading/Retraction

**Figure 6:**
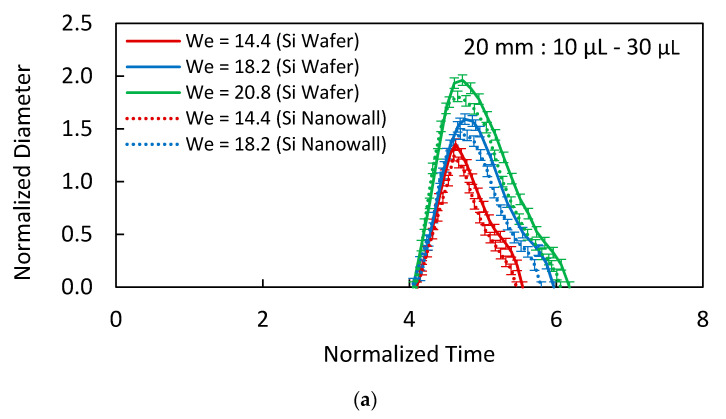

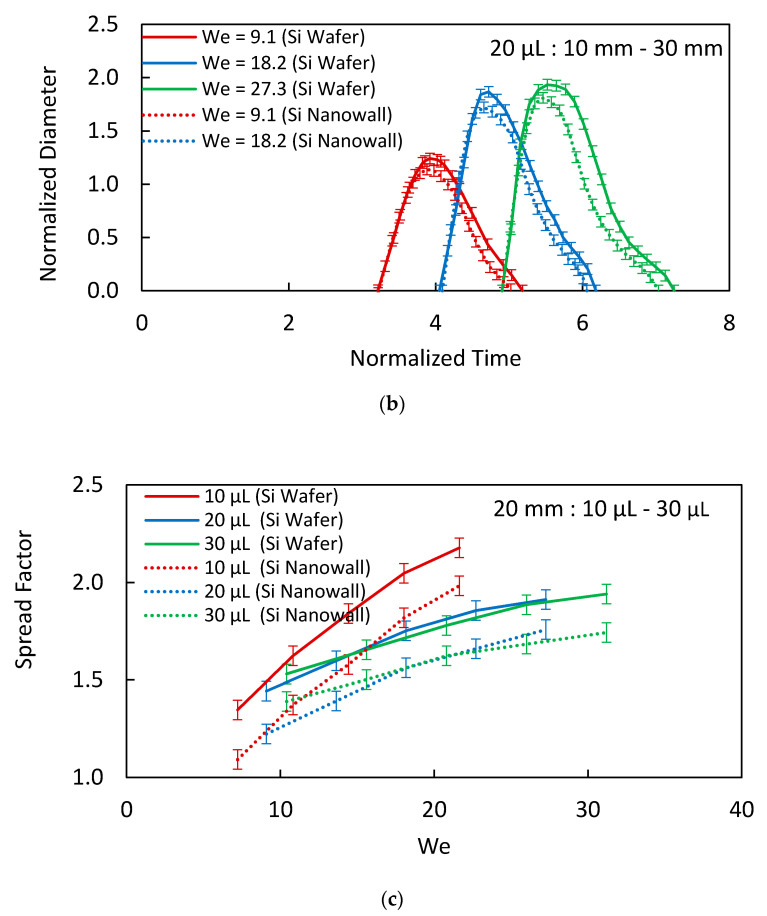
(**a**) Normalized spreading diameter variation with normalized time for 20 mm height and 10–30 µL droplet volumes. (**b**) Normalized spreading diameter variation with normalized time for 20 µL and 10–30 mm impact heights. (**c**) Spread factor variation with Weber number for 20 mm height and 10–30 µL droplet volumes.

The wetting diameter of the droplet on the impacting surface changes with time. In this case, the surface texture (nanopore sites created by the nanowalls) plays an important role in this change. Figure 6a shows normalized droplet wetting diameter of the spreading of various volumes droplet with the normalized time for hydrophobized textured and hydrophobized as-received smooth silicon-wafer surfaces. The droplet wetting diameter is normalized by the droplet diameter before the impact, i.e., ${\bar{D}}_{imp}=\frac{D_{imp}}{D_{0}}$, here ${\bar{D}}_{imp}$ is the normalized impacted droplet diameter, $D_{imp}$ is the instant droplet wetting diameter on the impacted surface, and $D_{0}$ is the droplet diameter before the impact. In addition, the normalized time resembles the ratio of the duration of the droplet spreading and the retraction over the inertial-capillary time ($\tau_{c}$), i.e., $\bar{t}=\frac{t}{\tau_{c}}$, here $\tau_{c}$ is the inertial-capillary timescale ($\tau_{c}=\sqrt{\frac{\rho D_{o}^{3}}{8\gamma }}$). It is worth mentioning that the initial stage of the impacting droplet can be assessed by the inertial-capillary timescale [39]. The inertial-capillary timescale signifies the time of response of the impacting droplet as if the droplet is considered to be a spring with the coefficient of surface tension as the stiffness of the spring [40]. Since various volumes of the impacting droplet are incorporated in the analysis, $\tau_{c}$ is kept constant for consistency of comparison; in which case, *D_o_* corresponding to the minimum droplet volume (10 μL) is adopted. The normalized wetting diameter during the initial period ($\bar{t}$ = 0.5) of spreading becomes almost the same for the droplet impacting on hydrophobized textured and hydrophobized as-received smooth silicon surfaces. As the spreading progresses before reaching the maximum droplet spreading diameter, the difference between the diameters due to hydrophobized textured and hydrophobized as-received smooth surfaces become large, and the difference reaches the maximum at the end of the spreading period. However, the difference in the spreading diameter remains almost the same during the retraction period of the droplet on both surfaces. Hence, during the early spreading period, high pressure in the droplet fluid overcomes the pinning and frictional losses (due to shear at the droplet fluid and pores surface interface) created on both surfaces. Therefore, the influence of porous-like texture becomes almost insignificant on the spreading diameter in the early impacting period. As the droplet spreading comes close to the end of the spreading period, the droplet fluid pressure weakens, and the pinning and shear effect created by the texture becomes important on the droplet spreading. The combinations of inertia, shearing, and surface tension effects can be formulated via Ohnesorge number ($Oh=\frac{\mu }{\sqrt{\rho \gamma R_{H}}}$, where μ is the droplet fluid viscosity). It takes a value of about 1.9 × 10^−3^ for impacting droplets, which is considerably small. Hence, the inertial and surface tension forces become more important on the droplet spreading as compared to the interfacial shearing effects. Increasing the impact height of the droplet increases the droplet inertia force, which enhances the spreading diameter of the droplet on the impacted surface. The energy losses related to the impacting droplet during the spreading and retraction periods, due to fluid shear, can be approximated by the following:

(7) $$W_{visc}=\frac{\pi }{3}\rho v_{i}^{2}D_{0}D_{max}^{2}\frac{1}{\sqrt{Re}}\left(\varphi +1\right)$$

where $W_{visc}$ is the work of viscous dissipation, *D_max_* is the maximum spreading diameter of the droplet, Re is the Reynolds number of impacting droplets, $v_{i}$ is the velocity of the spreading droplet, and φ is the solid fraction of the impacted surface [41]. Moreover, the elastic response of the impacted surface contributes to energy dissipation during the droplet impact. This becomes particularly important for soft surfaces, such as PDMS [42,43]. The rebounding height of the droplet reduces notably by viscoelastic energy dissipation [38,39]. The Young’s modulus of PDMS ranges between 0.57 to 3.7 MPa [43,44], while it is 130 to 188 GPa for silicon [45]. The energy dissipation due to the viscoelastic effect of the impacted surface is considered to be smaller than the energy dissipation, due to viscous dissipation and the work performed under volume deformation during impact. Hence, it is neglected in the analysis. The hydrophobized as-received silicon-wafer surface can be considered to be smooth, and the solid fraction, φ, becomes zero. Therefore, the slip velocity generated on the smooth hydrophobic silicon surface lowers the work of viscous dissipation. However, for the textured silicon-wafer surface, the slip velocity varies because of the presence of nanoporous textures (due to nanowalls). This alters the work of viscous dissipation. Hence, the ratio of work of dissipation, due to hydrophobized etched over the hydrophobized as-received surfaces, becomes ~(1+φ). Moreover, the solid fraction can be expressed as $\varphi \sim \frac{w^{2}}{4a^{2}}$ for nanopores’ surfaces [36]: here, w is the width of the nanowall and 2a is spacing in between two consecutive nanowalls. For nanowalls’ pores sties, it yields almost 0.3. Hence, the work of viscous dissipation becomes almost 30% more for the hydrophobized textured surface than a hydrophobized smooth surface. This contributes to the maximum spreading diameter of the droplet; that is, the maximum spreading diameter is smaller for hydrophobized textured silicon surface. As the droplet impact height increases, so does the maximum spreading diameter. This can be seen from Figure 6b, in which the normalized spreading diameter with normalized time is shown for various impact heights for 20 μL of the droplet. An increase in the spreading diameter is mainly because of the inertial influence of the impacting droplet on the spreading rates, i.e., low value of Ohnesorge number (~1.9 × 10^−3^). The increase of the droplet diameter with time is in the form $\bar{t}$^4^ up to the dimensionless time ($\bar{t}$) of 4.5. As the time progresses further toward that corresponding to the maximum droplet spreading diameter, the droplet diameter increase becomes in the form of $\bar{t}$^2^. Hence, the droplet speeding is rapid in the initial period, and it becomes gradual toward the end of the spreading period. Moreover, the spreading factor ($\frac{D}{D_{o}}$, where D is the instant droplet diameter on the impacted surface and *D_o_* is the droplet diameter before impact) of the droplet is also shown in Figure 6c to demonstrate the influence of Weber number ($We=\frac{\rho V^{2}D_{o}}{\gamma }$) on the droplet spreading for three droplet volumes.

**Figure 7:**
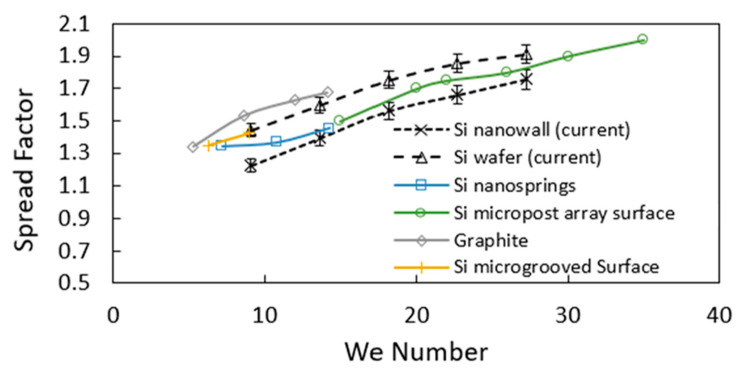
Comparison of spreading factor with Weber number for different surface types. □ Si nanosprings Adapted from Ref. [46]; ○ Si micropost array surface Adapted from Ref. [47]; ◊ Graphite Adapted from Ref. [48]; | Si microgrooved Surface Adapted from Ref. [13].

The spreading factor remains smaller for the hydrophobized textured surface (due to pinning and shear effects) than for the hydrophobized as-received smooth surface. Moreover, further assessment of the variation of spreading rate with the Weber number is made for various hydrophobized surfaces. Figure 7 depicts the comparison of the spreading factor with Weber number for various hydrophobic textures, including silicon nano-spring [46], silicon micro-post arrays [47], graphite [48], and silicon micro-grooved surfaces [13]. Hydrophobized nanowall surfaces result in the lowest spreading factor as compared to other surfaces considered. This behavior is related to the pinning influence of the closed-cell nanowalls structures on the surface that suppresses the droplet spreading on the surface during the impact. Moreover, the spreading factor increases with the Weber number. This indicates that the droplet inertial force contributes considerably to the droplet spreading. Increasing droplet volume enhances the inertial force and the Weber number. However, it also increases the droplet spreading diameter over the impacted surface. This causes increased pinning and the viscous forces acting on the spreading droplet while lowering the spreading factor (Figure 6c and Figure 7). The transition duration of the droplet on the impacted surface is shorter for the hydrophobized textured surface than that of the hydrophobized as-received smooth surface. This is true for all sizes and impact heights of the droplets (Figure 6b). The short duration of the droplet’s residence time on the impacting surface reveals that the work of deformation of the droplet because of shape change (volume deformation) becomes small (Appendix A). Hence, because of the work of volume deformation, the energy dissipated becomes small for the hydrophobic textured surface. In the case of the retraction period, the time taken for the droplet retracting to the initial wetting diameter is much larger than that of the spreading period. This is mainly because of the energy losses associated with impacting droplets (Appendix A), i.e., $W_{def}+W_{visc}+W_{add}$: here, $W_{def}$ is work performed during volumetric deformation of the droplet (droplet shape change), $W_{visc}$ viscous energy dissipation, and $W_{add}$ is the work performed against pinning. The droplet transitional velocity in the retraction period becomes smaller than the spreading period. Hence, the time taken for the rebounding of the droplet becomes long as the droplet-impact height increases (Figure 6b), which is also true for the case as the droplet volume increases. However, the difference between the retraction period of the droplet on hydrophobized textured and hydrophobized as-received smooth surfaces become small as the droplet volume increases while keeping the droplet impact height the same. This indicates that the droplet inertia has a critical influence on the retraction period compared to pinning and viscous dissipation alone.

### 3.4. Droplet Rebounding on Nanowall Textures

**Figure 8:**
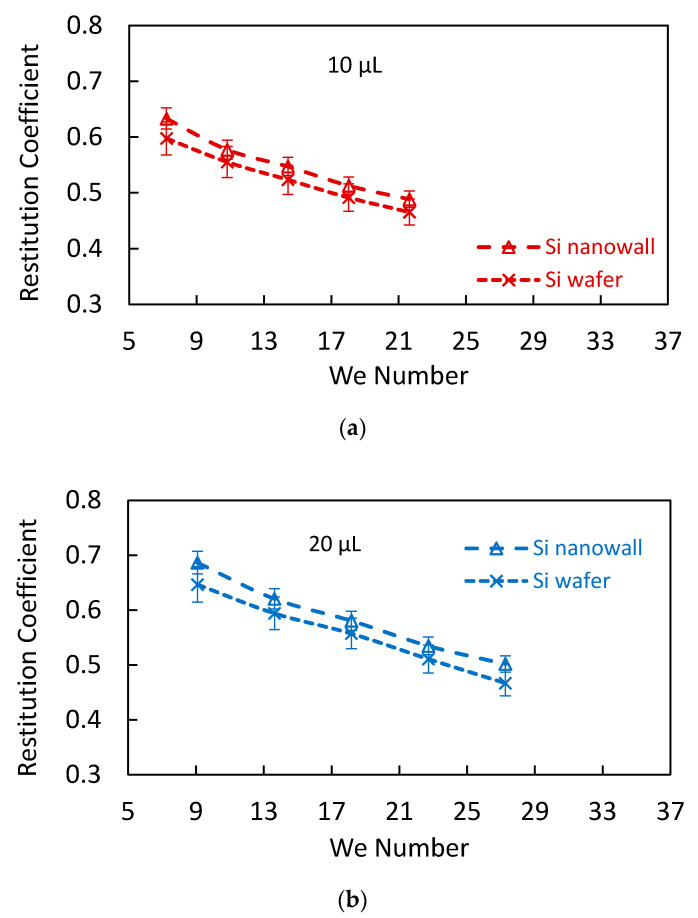

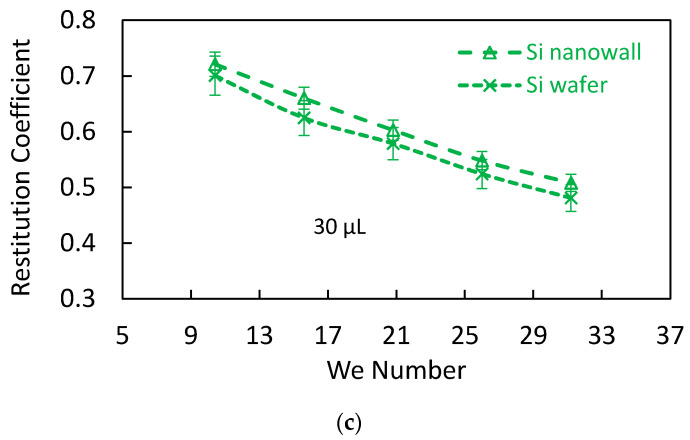
Restitution coefficient with Weber number for f: (**a**) 10 µL, (**b**) 20 µL, and (**c**) 30 µL droplets impacting on hydrophobized as-received silicon and hydrophobized etched silicon wafers.

Once the retraction period of the droplet ends, the droplet undergoes rebounding from the impacted hydrophobic surface. Figure 8 shows the restitution coefficient of the droplet with Weber number for the cases of hydrophobized textured and hydrophobized as-received smooth surfaces. The influence of three droplet volumes on the restitution coefficient is incorporated in Figure 8. The restitution coefficient is the measure of kinetic energy after and before the impacting droplet ($e=\sqrt{\frac{Kinetic\;Energy_{After\;Impact}}{Kinetic\;Energy_{Before\;Impact}}}$). The restitution coefficient remains high for the low values of the Weber number. This indicates that energy dissipated during the spreading and retraction periods becomes small as the Weber number reduces. It is worth mentioning that the Weber number is proportional to the droplet diameter and square of the droplet velocity. The hydrophobized textured (etched) surface results in larger values of the restitution coefficient as compared to that of the hydrophobized as-received smooth surface. This is related to the total energy dissipated during the droplet residency on the impacted surface. In addition, the air trapped within the nanowall structures is compressed by the impacting droplet fluid, and it acts as a compressed air cushion at the interface of the droplet fluid and the top surface of the nanowalls. This, in turn, contributes to the ease of droplet retraction on the impacted surface while reducing the pinning forces at the interface. Hence, the energy stored during compression of the trapped air contributes to the attainment of low energy dissipation in the droplet retraction cycle (Appendix A); that is, compressed trapped air creates a springboard effect on the rebounding droplet. It is worth mentioning that the penetration of droplet fluid in the nanowall cells results in the maximum droplet meniscus height (*δ*) of about 40 nm (Appendix A), which causes a pressure rise in the trapped air inside the nanowall cells. The droplet meniscus height enlarges with droplet size, which further increases the trapped air pressure in the cells. This gives rise to an increased restitution coefficient with increasing droplet volume. In addition, as the volume increases, the droplet spreading diameter on the impacted surface increases (Figure 6a), which enhances the coverage area of the trapped area at the interface. This improves the cushion effect of the compressed air at the interface while reducing energy dissipation during the droplet retraction period. However, as the Weber increases further beyond 30, the restitution coefficient reduces significantly, due to the droplet breakup into two secondary droplets; hence, only partial rebound is realized.

**Figure 9:**
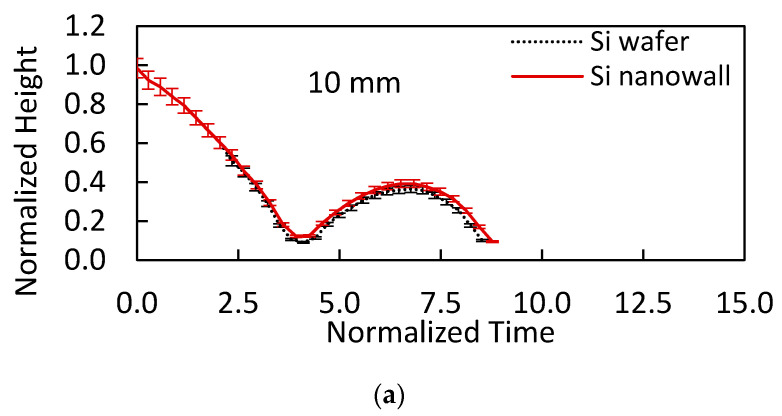

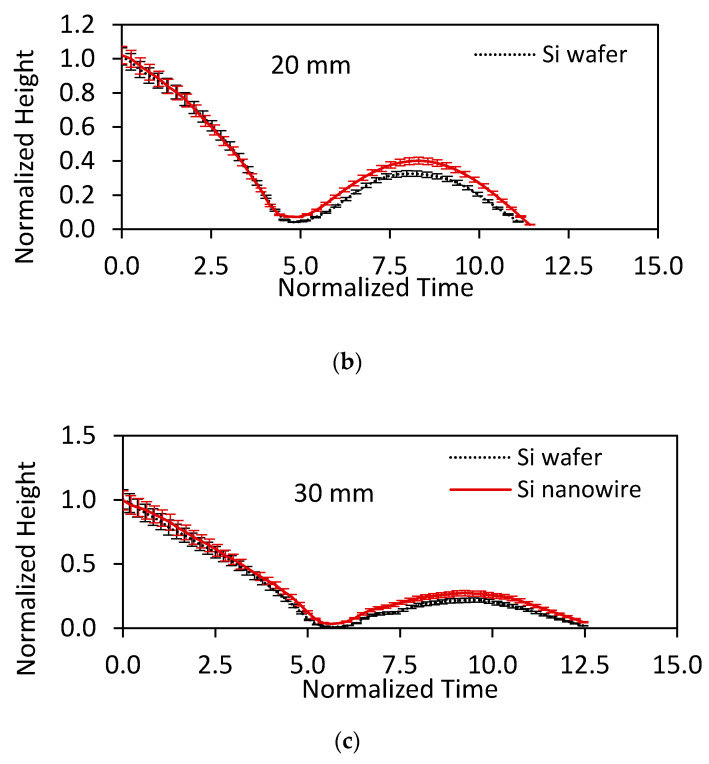
Rebound heights with normalized time for 20 µL volume droplet impacting on hydrophobized as-received silicon and hydrophobized etched silicon wafers from initial heights of (**a**) 10 mm, (**b**) 20 mm, and (**c**) 30 mm.

Figure 9 shows the droplet height with time during the impact and rebound periods for hydrophobized textured and as-received smooth surfaces. It is worth mentioning that the droplet height is normalized by the initial droplet impact height (*h_o_*). Since the initial impact heights of the droplet for hydrophobized textured and as-received surfaces are the same, only the rebound height of the droplet changes for both surfaces. The maximum rebound height of the droplet is larger for the hydrophobized textured surface than that corresponding to the hydrophobized as-received smooth surfaces for all initial impact heights. This indicates that the energy dissipated by the impacting droplet over the hydrophobized etched surface is less than the energy dissipation over the hydrophobized as-received smooth surface. In addition, small work of volume deformation, due to short spreading and retraction periods, and the trapped air spring-back effect contribute to the rebound height of the droplet.

## 4. Conclusions

Impacting-water-droplet behavior on the hydrophobized textured (nanowires/nanowalls) silicon wafer surface was examined. The controlled etching of the silicon wafer resulted in closely oriented nanowalls cells of about 130 nm in spacing and about 10.45 μm in length (height) on the wafer surface. In addition, the nano and sub-nano porous sites were observed on the top surface of the nanowall structures. Textured surfaces were hydrophobized to obtain the droplet contact angle of about 158° ± 2° with a hysteresis of 4° ± 1°. The findings revealed that the droplet spreading factor over the nanowalls textured hydrophobic surfaces is less than those of hydrophobized as-received silicon, hydrophobized graphite, micro-grooved, and nano-springs surfaces. This behavior is attributed to droplet fluid pinning over the nanowall textured surface under the surface tension influence. In this case, the droplet fluid adhesion lowers the spreading rate of the droplet over the surface. The rate of spreading on the hydrophobized nanowalls’ surface is related to the dimensionless time of $\bar{t}$ ($\bar{t}=\frac{t}{t_{c}}$, *t_c_* being the capillary time) in the early spreading cycle. As the spreading period increases, the rate of spreading becomes proportional to $\bar{t}$^4^. The retraction period of the impacted droplet on the hydrophobic nanowalls surface remains shorter than its counterpart for the hydrophobized as-received smooth surface. It becomes more apparent for the large size droplets with high impact heights. This is mainly related to the impacting droplet potential energy, which becomes considerably larger than the energy dissipation, due to the work performed against volume deformation and pinning during the retraction period. After spreading, the maximum droplet diameter becomes smaller for the hydrophobized nanowalls surface than for the received smooth surface. Similarly, the restitution coefficient and the droplet rebound height remain large for the nanowall textured surface, and this becomes more apparent for the large droplet volumes with high impact heights. In this case, trapped air inside the nanowall cells acts as an air cushion lowering the energy dissipation due to pinning and interfacial shear. In addition, the rise of pressure in the trapped air during the droplet fluid penetration acts as a springboard effect contributing to the rebound height of the droplet. The present study covered a detailed analysis of liquid droplets impacting hydrophobized nanowalls surfaces and provided information on impacting droplet characteristics toward designing nanowall hydrophobic surfaces.

## Figures and Tables

**Figure A1:**
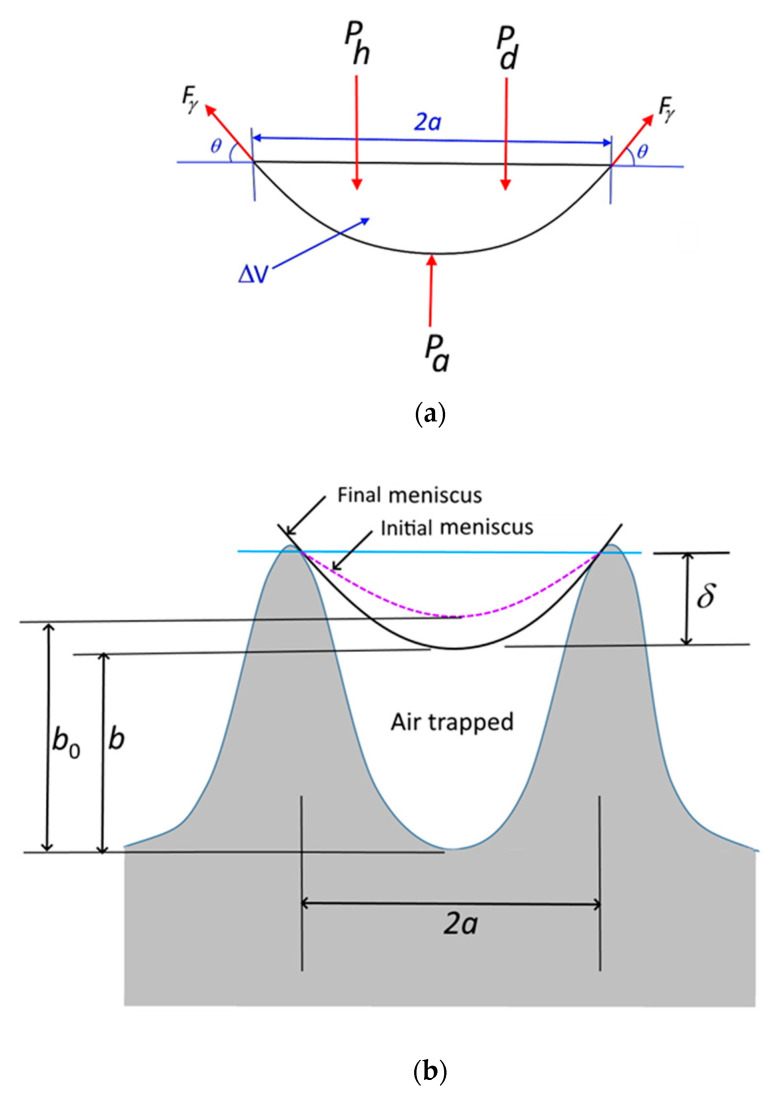
A schematic view of the nanostructured surface: (**a**) forces acting at the droplet meniscus across texture gap and (**b**) droplet meniscus across texture gap before and after the impact of the droplet.

## Data Availability

The data presented in this study are available upon request from the corresponding author.

## Appendix A. Analysis of Impacting Droplet Characteristics

Although the analysis related to the inertia-dominated drop collision and spreading of viscous film over the impacted area was presented earlier [33], the arguments are valid for high Weber and high Reynolds numbers of the impacting droplet. Moreover, in the analysis, axisymmetric droplet spreading is considered, and it can limit the utilization of the solution of the resulting equation for non-uniformly textured hydrophobic surfaces. This is because the non-axisymmetric spreading of the droplet takes place over the non-uniformly textured surfaces. In addition, in the analytical solution, the radial pressure gradient and slip on the impacted surface, which are the cases for droplet spreading over the hydrophobic surfaces, are omitted to obtain a self-similar solution to the problem [33]. On the other hand, the nanowall structures have irregular topological geometry, and it is difficult to incorporate the three-dimensional formulation in terms of energy balance. Consequently, the energy balance satisfying the low Reynold number impacting droplet is presented while using the lump analysis. Hence, the impacting droplet initial energy can be expressed as follows [2]:

(A1) $$E_{pot}+S_{1}=S_{2}+\Delta W+E_{reb}=\rho \frac{4}{3}\pi R^{3}v_{i}^{2}$$

where $E_{pot}$ is impacting droplet potential energy, $E_{reb}$ is rebounding droplet potential energy, $S_{1}$ is initial surface energy, $S_{2}$ is surface energy at maximum spread, ΔW net energy gain/loss during spreading, ρ is the fluid density, $R_{0}$ is droplet initial radius, and $v_{i}$ is impact velocity.

The net energy gain/loss is composed of several components, which are as follows:

(A2) $$\Delta W=W_{def}+W_{visc}+W_{fric}+W_{spring}$$

where $W_{def}$ is the work performed due to droplet deformation during the transition on the impacted surface, $W_{visc}$ viscous energy loss, $W_{fric}$ is the energy loss against friction, and $W_{spring}$ is energy gain during the recoiling of droplets due to the spring-back effect resulting from the formation of meniscus within the nanowalls cells.

The energy terms can be expressed as follows [39]:

(A3) $$S_{1}=\pi D_{0}^{2}\gamma$$

(A4) $$S_{1}=\frac{\pi }{4}D_{max}^{2}\gamma \left(1-cos\theta_{a}\right)$$

where $D_{max}$ is maximum droplet diameter, $D_{0}$ is droplet initial diameter, γ is surface tension, and $\theta_{a}$ is advancing contact angle. The work of deformation can be approximated as follows:

(A5) $$W_{def}=2\forall_{0}\left(p_{i}-p_{r}\right)$$

where $\forall_{0}$ is droplet volume, $p_{i}=\frac{4\gamma }{D_{0}}+\frac{1}{2}\rho v_{i}^{2}$ is droplet pressure unset of impact, $p_{r}=\frac{2\gamma \left(t_{h}+D_{ret}\right)}{t_{h}D_{0}}$ is droplet pressure during retraction stage, $t_{h}=\frac{4\forall_{0}}{\pi D_{ret}^{2}}$ is maximum droplet height (thickness) during spreading, and $D_{ret}$ is the diameter of the droplet after the retraction period.

The viscous work becomes the following:

(A6) $$W_{visc}=\frac{\pi }{3}\rho v_{i}^{2}D_{0}D_{max}^{2}\frac{1}{\sqrt{Re}}\left(\varphi +1\right)$$

where φ is the solid fraction of the impacted surface, φ~0 for as-received silicon wafer and $\varphi \sim \frac{\pi w^{2}/4}{\pi a^{2}}$ for silicon nanowall cells, w is the width of nanowalls, and 2a is spacing between the walls [39]. The work of friction can be expressed as follows:

(A7) $$W_{fric}\;\sim \;0\;\mathrm{for}\;\mathrm{as}-\mathrm{received}\;\mathrm{silicon}\;\mathrm{wafer}\;\mathrm{and}\;W_{fric}=\frac{\alpha \mu \rho D_{max}^{4}v_{i}^{3}}{48\gamma D_{0}\ln\left(\frac{2a}{w}\right)}\;\mathrm{for}\;\mathrm{silicon}\;\mathrm{nanowall}\;\mathrm{cells}$$

where α is a relaxation factor and μ is dynamic viscosity [49].

When the droplet impacts, partial impalement of the droplet (or formation of meniscus) inside the nanopores gives rise to the spring-back effect. Hence, the air is trapped in between the droplet interface and the nanostructured surface, and its spreading is inhibited. In this case, the droplet behaves similar to an elastic body such that a vertical restoring force contributes to its rebounding kinetic energy. The higher the volumetric change due to impalement, the larger the magnitude of the lifting force contributing to the rebound height of the droplet, i.e., it mimics the elastic behavior of springs (F~kx) [25]. Hence, the work contributing to spring-back yields the following: $W_{spring}=\Delta P_{a}\Delta V_{a}=F_{T}\delta =kx\cdot \delta$ is an additional energy gain by the droplet due to the movement of droplet meniscus within the air entrapped enclosed by the silicon nanopores. Moreover, $\Delta P_{a}$ and $\Delta V_{a}$ are the corresponding change in pressure and volume of the entrapped air.

The coefficient of restitution can be expressed as follows:

(A8) $$e=\frac{v_{reb}}{v_{i}}=\sqrt{\frac{h_{reb}}{h_{i}}}=\sqrt{\frac{E_{reb}}{E_{pot}}}=\sqrt{1-\frac{\Delta W}{\rho \frac{4}{3}\pi R^{3}v_{i}^{2}}}$$

Dimensionless quantities:

$$Re=\frac{\rho v_{i}D_{0}}{\mu },\;Bo=\frac{\gamma_{f}R_{0}^{2}}{\gamma },\;We=\frac{\rho v_{i}^{2}D_{0}}{\gamma },\;\beta =\frac{D}{D_{0}},\;\bar{t}=\frac{t}{\tau_{c}},\;{\bar{t}}_{h}=\frac{t_{h}}{D_{0}},\;{\bar{D}}_{wet}=\frac{D_{wet}}{D_{0}},\;\bar{h}=\frac{h}{h_{0}},\;\bar{v}=\frac{v}{v_{i}}$$

### Spring-Back Phenomenon during Droplet Impact on Silicon Nanowall Cells

During the droplet impact on the surface of the silicon nanowall cells, the air is trapped in between the droplet interface and the nanostructured surface. The droplet meniscus height over a single closed packed nanopore can be obtained by the balance of forces acting at the interface. When the droplet impacts, the arc is formed at the droplet meniscus inside the nanopores giving rise to the momentary compression–suction cycle within the entrapped air. Hence, the droplet behaves similar to an elastic body, and a vertical restoring force is developed during the rebounding stage of the droplet. The higher the volumetric change, the higher the magnitude of the lifting force mimicking the spring-back effect.

In the case of the nanowall cell surfaces, the texture composes of some closed packed gaps. The volume of air trapped in a nanowall cell can be considered to be a half ellipsoid. Hence, the height of the meniscus across a single cell can be depicted schematically in Figure A1. The droplet meniscus height after impact can be evaluated after adopting the vertical force balance. When considering Figure A1, we see that the balance of forces in the vertical direction yields the following:

(A9) $$\rho g\pi a^{2}h_{d}+\rho g\Delta V+P_{eff}\pi a^{2}=F_{\gamma }sin\theta$$

where ρg is the specific weight, $h_{d}$ represents the height of droplet at the onset of rebounding, Fγ is the surface tension force, ΔV corresponds to the inflection volume, and $P_{eff}=\frac{1}{2}\rho v_{eff}^{2}$ is effective impact pressure. Hence, Equation (A9) can be expressed as follows:

(A10) $$g\pi a^{2}h_{d}+\frac{1}{2}\rho g\frac{4\pi }{3}a^{2}\delta +p_{eff}\pi a^{2}=2\pi a\gamma sin\theta$$

Since $sin\theta \approx \sqrt{\frac{\delta^{2}}{\left(a^{2}+\delta^{2}\right)}}$, when we divide by $\rho g\pi a^{2}$, it yields the following:

(A11) $$\frac{2}{3}\delta -\frac{2\gamma \delta }{\rho ga}\sqrt{\frac{\delta^{2}}{\left(a^{2}+\delta^{2}\right)}}+h_{d}+\frac{p_{eff}}{\rho g}=0$$

The solution of Equation (A11) yields the functional relation between the droplet meniscus height (δ) and fluid properties, as well as the lateral distance across the meniscus of the droplet in two consecutive nanowalls.

By considering the entrapped air as an ideal gas, an increase of pressure due to the compression of entrapped air can be formulated as follows:

(A12) $$\Delta P_{a}=-mRT\int_{V_{1}}^{V_{2}}\frac{dV}{V^{2}}$$

where R is the gas constant of air, T is temperature, m is the air mass, and *V* is the volume.

Moreover, consider ΔV as the half volume of an ellipsoid; the change in volume of the entrapped air is related to the meniscus height, b, which can be expressed as follows:

(A13) $$dV_{a}=\frac{4}{6}\pi a^{2}db$$

Hence, Equation (A16) can be expressed as follows:

(A14) $$\Delta P_{a}=-mRT\int_{V_{1}}^{V_{2}}\frac{dV}{V^{2}}=-mRT\int_{b_{0}}^{b}\frac{\frac{4}{6}\pi a^{2}db}{{\left(\frac{4}{6}\pi a^{2}b\right)}^{2}}=\frac{-6mRT}{\pi a^{2}}\left(\frac{1}{b_{0}}-\frac{1}{b}\right)$$

The pressure force generated in a single packed texture gap is as follows:

(A15) $$F=\pi a^{2}\Delta P_{a}$$

The number of cells on the nanowalls’ silicon surface can be used to find the force of pressure on the meniscus. Therefore, approximately, pressure force becomes as follows:

(A16) $$F=n\pi a^{2}\Delta P_{a}$$

where *n* represents the number of cell gaps on the nanowalls silicon surface. Rearranging Equations (A15) and (A16) gives rise to the force of pressure, which yields the following:

(A17) $$F_{T}=6nmRT\left[\frac{1}{b}-\frac{1}{b_{0}}\right]$$
